# Supplementary material for: Substantial somatic genomic variation and selection for BCOR mutations in human induced pluripotent stem cells
Source: Nat Genet. 2022 Aug 11;54(9):1406–16. doi: 10.1038/s41588-022-01147-3 (PMC9470532; doi:10.1038/s41588-022-01147-3)
Supplement: Supplementary file 1 — Supplementary note and Supplementary Figures 1–5. [file 41588_2022_1147_MOESM1_ESM.pdf]

nature portfolio

https://doi.org/10.1038/s41588-022-01147-3

Supplementary information

Substantial somatic genomic variation and selection for *BCOR* mutations in human induced pluripotent stem cells

In the format provided by the authors and unedited

Supplementary Information

Supplementary Note

The insignia cohort comprised of erythroblast-derived B-hiPSCs created from 78 individuals: 53 patients with inherited DNA repair defects including Oculomotor apraxia type 2 (*AOA2*), ataxia telangiectasia (*ATM*), selenoprotein deficiency (*SECISBP2)*, Lynch Syndrome, Xeroderma Pigmentosum (*XPA*, *XPC, XPD, XPE, XPG* and *XPV*), homologous recombination deficiency (*BRCA1* and *BRCA2*), constitutional mismatch repair deficiency (*PMS2* and *MSH6*), five patients with exposure to environmental agents (chemotherapy at young age or fetal exposure to maternally-ingested valproate) and 20 healthy controls (Table S8).

Supplementary Figures

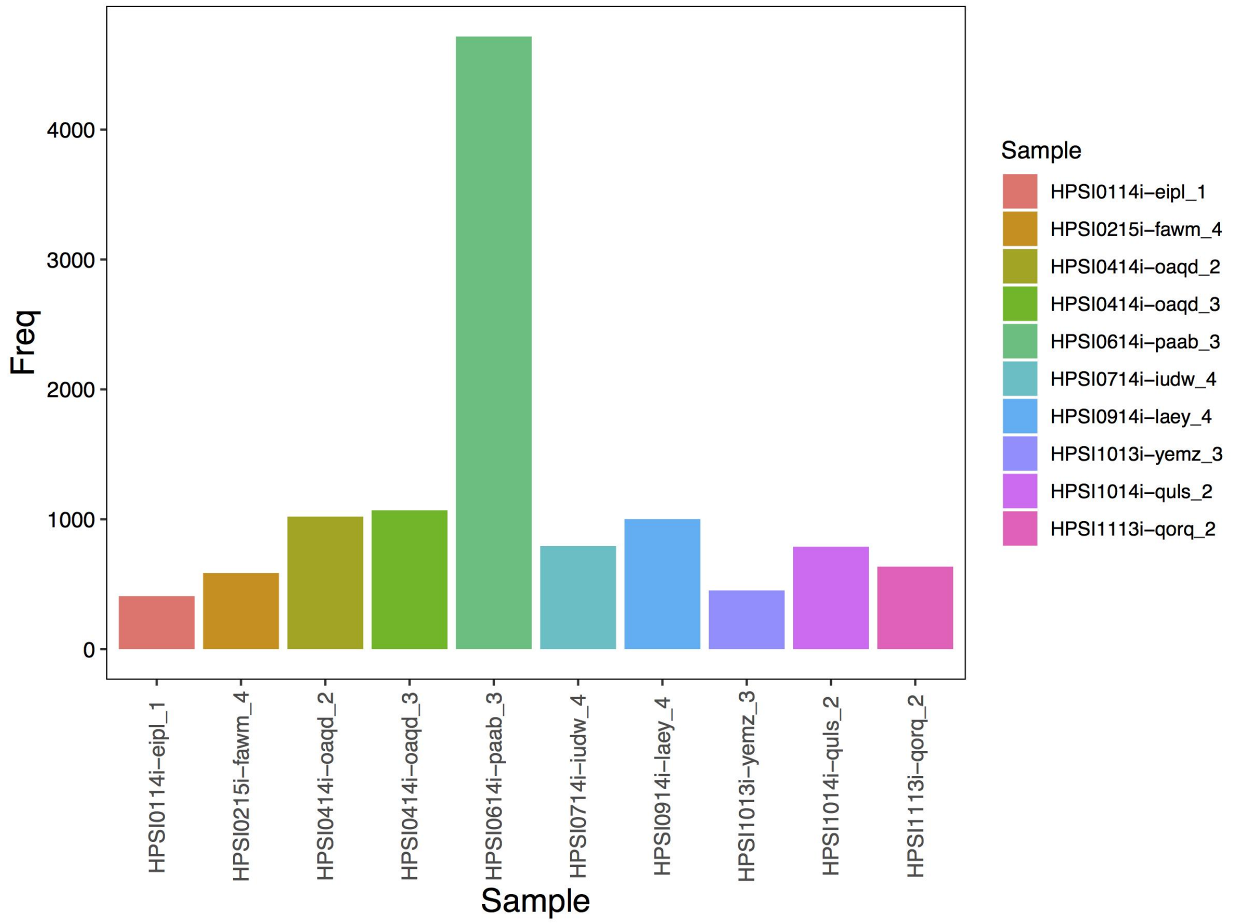

Supplementary Fig. 1. Shared mutations in F-hiPSCs and fibroblasts. Histogram showing number of substitutions that were removed from iPSCs by using fibroblast as “normal” for ten HipSci samples from Figure 1.

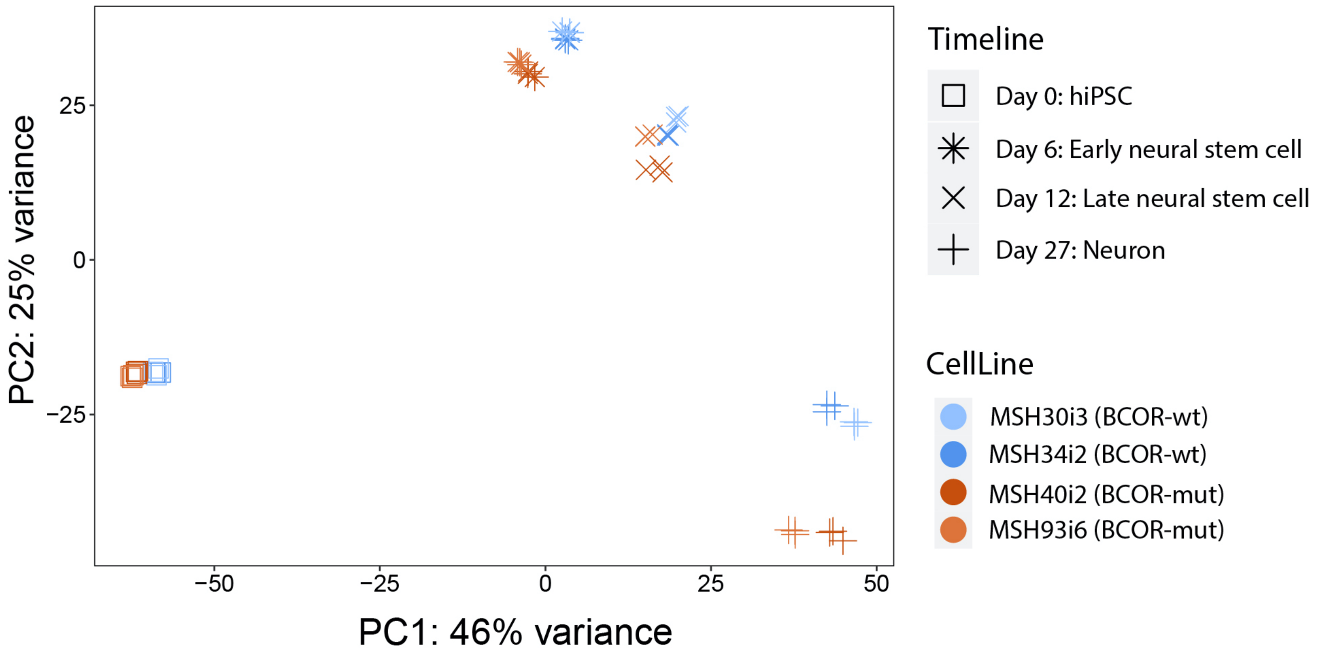

Supplementary Fig. 2. Principal Component (PC) Analysis of RNA sequencing data. PC analysis of RNA-seq data shows transcriptomic differences in both BCOR-mut lines compared to both BCOR-wt samples, across the neural differentiation stages.

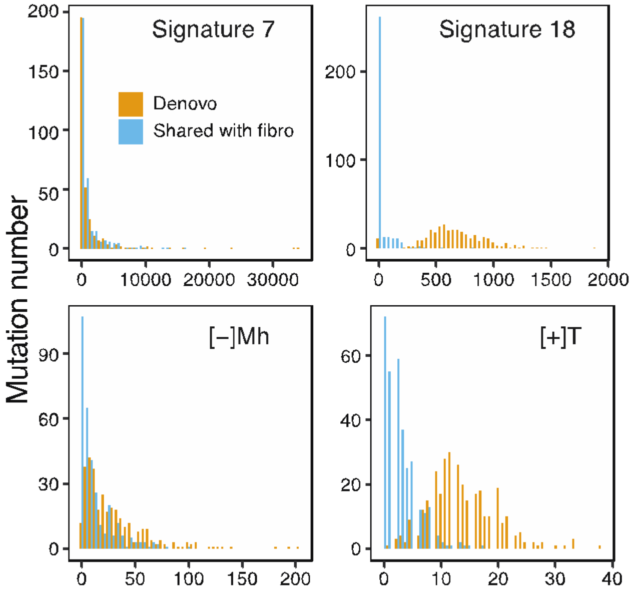

Supplementary Fig. 3. Histogram of shared and private (de novo) mutations for signature 7 (UV), signature 18 (oxidative damage), [-]Mh and [+]T. Signature 18 was prevalent in de novo variants, in contrast to shared variants.

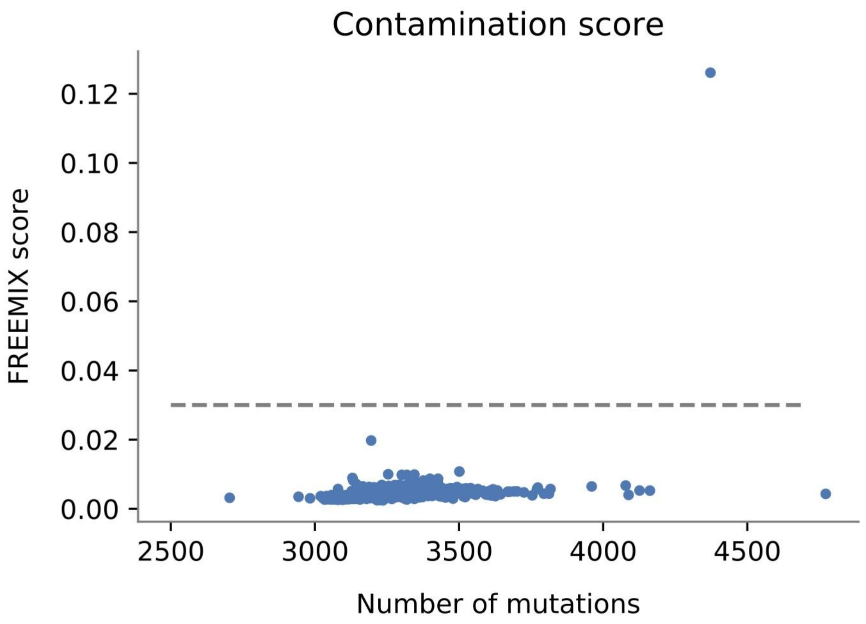

Supplementary Fig. 4. Contamination score of cell lines. There is no evidence of contamination except for one cell line and there is no correlation between the number of mutations and the FREEMIX score (R^2=0.1). The dashed line at 0.03 is the threshold suggested by VerifyBamID to accept or potentially flag the sample as contaminated. The outlier cell line (HPSI0913pf-coyi) was removed from analysis.

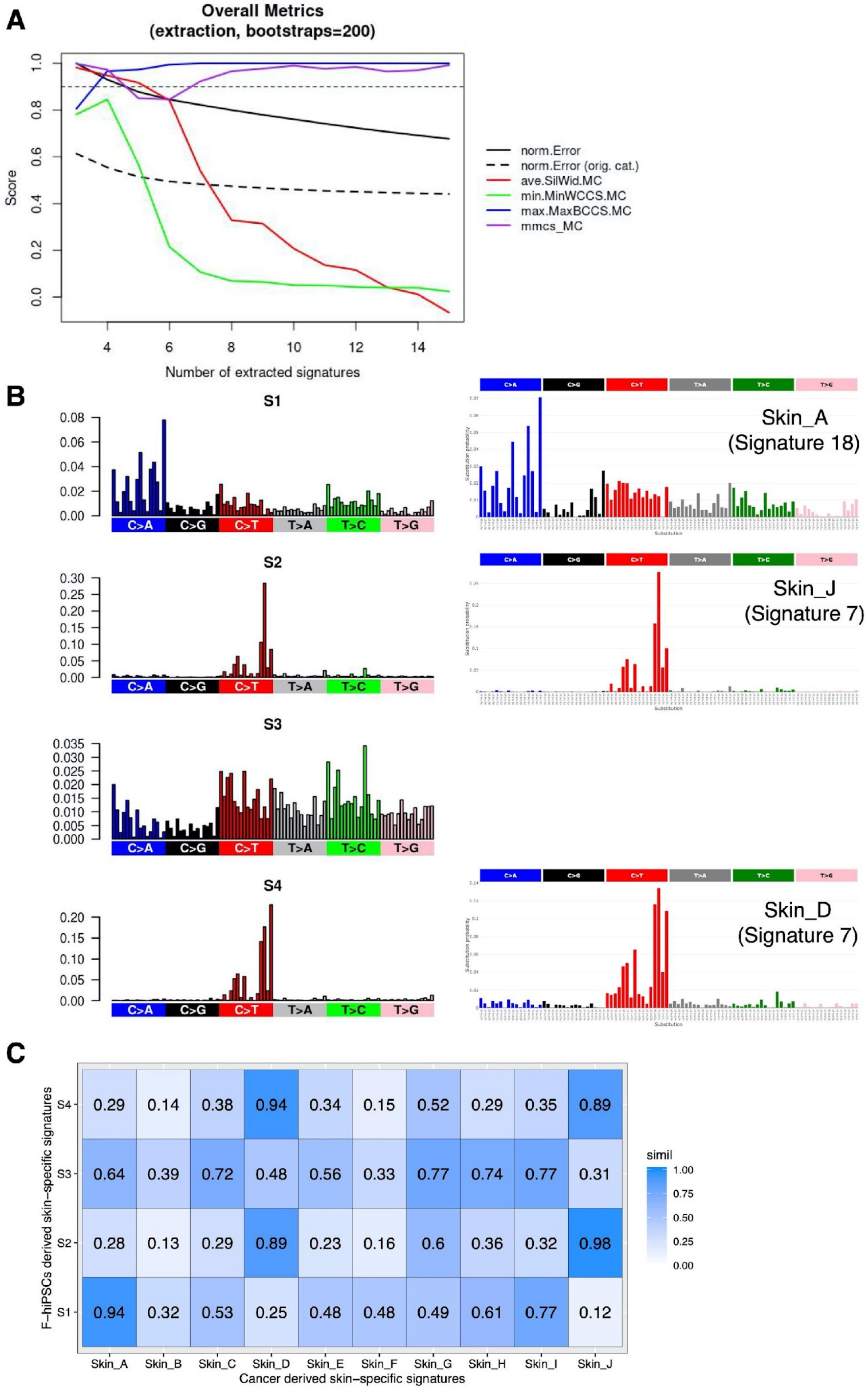

Supplementary Fig 5. ***De novo*** **extraction on 324 skin-derived WGS hiPSCs from the HipSci project.** (A) Metrics for selecting the optimal number of signatures. (B) Four mutational signatures extracted from this data set. Profiles of similar skin cancer derived signatures are shown. (C) Cosine similarities between F-iPSCs signatures and skin cancer derived signatures. S2 and S4 are most similar (cossim: 0.94-0.98) to UV-associated mutational signatures, Skin_J and Skin_D (signature 7), respectively. S1 is most similar to Skin_A (signature 18), the culture signature (cossim: 0.94). S3 does not

show high similarity to any skin-specific signatures (cossim <0.8), but also has very low probabilities for all 96 channels (note y-axis values are very small), and the relatively featureless profile would suggest that it is likely to be “noise”. This is not uncommon in signature extractions.
