# Supplementary material for: Substantial somatic genomic variation and selection for BCOR mutations in human induced pluripotent stem cells
Source: Nat Genet. 2022 Aug 11;54(9):1406–16. doi: 10.1038/s41588-022-01147-3 (PMC9470532; doi:10.1038/s41588-022-01147-3)
Supplement: Supplementary file 2 — Reporting Summary [file 41588_2022_1147_MOESM2_ESM.pdf]

nature portfolio

Corresponding author(s): Dr Serena Nik-Zainal

Last updated by author(s): Mar 29, 2022

Reporting Summary

Nature Portfolio wishes to improve the reproducibility of the work that we publish. This form provides structure for consistency and transparency in reporting. For further information on Nature Portfolio policies, see our Editorial Policies and the Editorial Policy Checklist.

Statistics

For all statistical analyses, confirm that the following items are present in the figure legend, table legend, main text, or Methods section.

n/a	Confirmed	
☐	☒	The exact sample size (*n*) for each experimental group/condition, given as a discrete number and unit of measurement
☐	☒	A statement on whether measurements were taken from distinct samples or whether the same sample was measured repeatedly
☐	☒	The statistical test(s) used AND whether they are one- or two-sided<br>*Only common tests should be described solely by name; describe more complex techniques in the Methods section.*
☒	☐	A description of all covariates tested
☒	☐	A description of any assumptions or corrections, such as tests of normality and adjustment for multiple comparisons
☐	☒	A full description of the statistical parameters including central tendency (e.g. means) or other basic estimates (e.g. regression coefficient) AND variation (e.g. standard deviation) or associated estimates of uncertainty (e.g. confidence intervals)
☐	☒	For null hypothesis testing, the test statistic (e.g. *F*, *t*, *r*) with confidence intervals, effect sizes, degrees of freedom and *P* value noted<br>*Give P values as exact values whenever suitable.*
☒	☐	For Bayesian analysis, information on the choice of priors and Markov chain Monte Carlo settings
☒	☐	For hierarchical and complex designs, identification of the appropriate level for tests and full reporting of outcomes
☐	☒	Estimates of effect sizes (e.g. Cohen's *d*, Pearson's *r*), indicating how they were calculated

Our web collection on statistics for biologists contains articles on many of the points above.

Software and code

Policy information about availability of computer code

Data collection	CaVEMan (http://cancerit.github.io/CaVEMan/) (Jones et al., 2016), Pindel (http://cancerit.github.io/cgpPindel) (Raine et al., 2015). The code of bespoke software is on github: https://github.com/dg13/ips-seq.
Data analysis	All code used for analysis are detailed in the Methods section. The code of statistical analysis and figures is on github: https://github.com/Nik-Zainal-Group/hiPSCs_BCOR.git. dN/dS ratios were calculated using dNdScv R package (https://github.com/im3sanger/dndscv)

For manuscripts utilizing custom algorithms or software that are central to the research but not yet described in published literature, software must be made available to editors and reviewers. We strongly encourage code deposition in a community repository (e.g. GitHub). See the Nature Portfolio guidelines for submitting code & software for further information.

Data

Policy information about availability of data

All manuscripts must include a data availability statement. This statement should provide the following information, where applicable:

- Accession codes, unique identifiers, or web links for publicly available datasets
- A description of any restrictions on data availability
- For clinical datasets or third party data, please ensure that the statement adheres to our policy

Links to raw sequencing data produced in this study are available from the HipSci project website (www.hipsci.org). Raw data are deposited in the European Nucleotide Archive (ENA) under the following accessions: ERP006946 (WES, open access samples) and ERP017015 (WGS, open access samples), and in the European Genotype-Phenotype Archive under accession EGAS00001000592 (WES, managed access samples). The open access data samples are freely available to download, while the managed access data are available following a request and a data access agreement. The variant call sets are deposited at Mendeley: https://data.mendeley.com/datasets/6rfc2xrnyd/1

Field-specific reporting

Please select the one below that is the best fit for your research. If you are not sure, read the appropriate sections before making your selection.

☒ Life sciences ☐ Behavioural & social sciences ☐ Ecological, evolutionary & environmental sciences

For a reference copy of the document with all sections, see nature.com/documents/nr-reporting-summary-flat.pdf

Life sciences study design

All studies must disclose on these points even when the disclosure is negative.

Sample size	The sample size was 555 human iPS cells lines from multiple cohorts including HipSci, one of the largest iPS cell repositories in the world. This was a systematic study on the mutational landscape of hiPSC WGS. We included as many iPSCs as possible and therefore maximized the sample size as much as possible.
Data exclusions	No data or cell lines were excluded.
Replication	The data analysis was replicated independently by co-authors. Some patients had multiple iPS cells lines generated and sequenced. Some B-hiPSCs were single-cell sub-cloned (outlined in Table S14). The differentiation and immunostaining experiments were performed in triplicates.
Randomization	There was no randomization as it was an observational and descriptive study on human iPS cells.
Blinding	This was not a clinical trial. The investigators were not blinded to allocation during experiments and outcome assessment.

Reporting for specific materials, systems and methods

We require information from authors about some types of materials, experimental systems and methods used in many studies. Here, indicate whether each material, system or method listed is relevant to your study. If you are not sure if a list item applies to your research, read the appropriate section before selecting a response.

Materials & experimental systems

n/a	Involved in the study	
☒	☐	Antibodies
☐	☒	Eukaryotic cell lines
☒	☐	Palaeontology and archaeology
☒	☐	Animals and other organisms
☐	☒	Human research participants
☒	☐	Clinical data
☒	☐	Dual use research of concern

Methods

n/a	Involved in the study	
☒	☐	ChIP-seq
☒	☐	Flow cytometry
☒	☐	MRI-based neuroimaging

Eukaryotic cell lines

Policy information about cell lines

Cell line source(s)	The cell lines were from HipSci and the Insignia projects. Some hiPSCs from S2 and S7 have been previously published (https://doi.org/10.1371/journal.pgen.1004432)
Authentication	The cell lines have been authenticated by HipSci and Insignia .
Mycoplasma contamination	All cell lines tested negative for mycoplasma.
Commonly misidentified lines (See ICLAC register)	There were no commonly misidentified lines.

Human research participants

Policy information about studies involving human research participants

Population characteristics	All participants were organ donors, volunteers via the NIHR Cambridge Bioresource or recruited for the Insignia project (doi: https://doi.org/10.1101/2020.08.04.234245) and appropriately consented. The exact age information of the human patient samples is not publicly available as the information could compromise privacy and lead to identification of individuals. The gender and genotype of the Insignia participants are as follows:<br><br>Samples Genotype Gender

MSH65 AOA2 Male
MSH69 AOA2 Male
MSH70 AOA2 Male
MSH80 AT Female
MSH11 ATM Female
MSH5 Chemo Female
MSH88 Chemo Female
MSH77 CMMRD Male
MSH94 CMMRD Female
MSH25 Control Female
MSH39 Control Female
MSH53 Control Female
MSH59 Control Male
MSH63 Control Male
MSH98 Control Male
MSH20 XPC Male
MSH40 XPC Male
MSH93 XPD Male
MSH95 XPD Female
MSH97 XPD Male
MSH43 XPG Male
MSH71 AOA2 Female
MSH68 AOA2 Male
MSH13 ATM Female
MSH29 Control Female
MSH90 Myelofibrosis Male
MSH3 PMS2 Female
MSH41 XPC Male
MSH67 AOA2 Male
MSH72 AOA2 Male
MSH14 ATM Female
MSH28 ATM Female
MSH35 ATM Female
MSH44 ATM Male
MSH48 ATM Male
MSH46 BLM Male
MSH73 BRCA1 Male
MSH75 BRCA1 Male
MSH74 BRCA2 Male
MSH76 BRCA2 Male
MSH89 CMMRD Female
MSH54 Cockaynes Female
MSH55 Cockaynes Female
MSH22 Control Male
MSH23 Control Female
MSH26 Control Female
MSH31 Control Male
MSH33 Control Female
MSH36 Control Female
MSH45 Control Female
MSH52 Control Female
MSH58 Control Female
MSH61 Control Male
MSH62 Control Female
MSH64 Control Female
MSH87 Control Female
MSH51 FVS Female
MSH56 FVS Male
MSH79 FVS Female
MSH47 Lynch Female
MSH60 Lynch Male
MSH78 Lynch Female
MSH57 RTS Female
MSH6 SECISBP2 Male
MSH8 SECISBP2 Male
MSH2 XPA Male
MSH86 XPB Female
MSH21 XPC Male
MSH42 XPC Female
MSH30 XPD Male
MSH37 XPD Male
MSH91 XPE Female
MSH27 XPG Female
MSH32 XPG Female
MSH34 XPG Male
MSH92 XPV Male

	MSH19 XPV Male<br>MSH85 XPV Female
Recruitment	Recruitment was voluntary for all research participants. We aimed to recruit as many patients with DNA repair deficiency as possible.
Ethics oversight	HipSci: REC 09/H0304/77, V2 04/01/2013, REC 09/H0304/77, V3 15/03/2013; Insignia: REC 13/EE/0302; organ donors: REC 09/H306/73)

Note that full information on the approval of the study protocol must also be provided in the manuscript.
